# Building the Future of Bioinformatics through Student-Facilitated Conferencing

**DOI:** 10.1371/journal.pcbi.1003458

**Published:** 2014-01-30

**Authors:** Kavisha Ramdayal, Miranda D. Stobbe, Tarun Mishra, Magali Michaut

**Affiliations:** 1 South African National Bioinformatics Institute, University of the Western Cape, Cape Town, South Africa; 2 Bioinformatics Laboratory, Academic Medical Center, University of Amsterdam, Amsterdam, The Netherlands; 3 Netherlands Bioinformatics Centre, Nijmegen, The Netherlands; 4 Bioinformatics Division, School of Bio Sciences and Technology, VIT University, Vellore, India; 5 Computational Cancer Biology, The Netherlands Cancer Institute, Amsterdam, The Netherlands; Princeton University, United States of America

## Abstract

Sharing results, techniques, and challenges is paramount to advance our understanding of any field of science. In the scientific community this exchange of ideas is mainly made possible through national and international conferences. Scientists have the opportunity to showcase their work, receive feedback, and improve their presentation skills. However, conferences can be large and intimidating for young researchers. In addition, for many of the more prestigious conferences, the very high number of submissions and low selection rate are major limitations to aspiring young researchers aiming to present their work to the scientific community. To improve student participation and proliferation of information, regional student groups have successfully organized conferences and symposia specifically aimed at students. This gives more students the opportunity to present their work and receive valuable experience and insight from peers and leaders in the field. At the same time, it is an ideal way for students to gain familiarity with the conference experience. In this paper, we highlight some of the benefits of participating in such student conferences, and we review the challenges we have encountered when organizing them. Both topics are illustrated in detail with examples from different ISCB Student Council Regional Student Groups.

## Introduction

Regional student groups (RSGs) affiliated with the Student Council (SC) of the International Society for Computational Biology (ISCB) have been working together to organize conferences and symposia around the world [1]–[4] with the aim of facilitating student participation and interactions [5].

We define a conference or symposium as a meeting in which participants with a shared interest discuss their work with each other in person or online. Typically a symposium is held over a single day, while a conference is held over several days. However, here we will refer to both collectively as conferences. The size of these events varies widely, from a small meeting with just a dozen participants to huge events with thousands of participants. Conferences usually focus on a lecture-based format where there is a single presenter and the rest of the audience listens (or takes a nap). This is in contrast to workshops, which, as the name already implies, require the participants to be more active. Some conferences encompass broad areas and topics, while others are themed or subcategorized into different research areas for specialized scientists.

## The Benefits of Student Conferences

Here we focus on conferences organized by and for students. These student conferences enable formal and informal introductions between young researchers in the field who can learn from each other and potentially make plans for collaboration on future projects. One key element that distinguishes these student events from regular conferences is that they provide a more relaxed atmosphere for students to present their work to their peers. Senior scientists are often invited to give keynote lectures and encouraged to stay for the day. In contrast to many international meetings, however, they tend to be more approachable during and after their presentations because fewer fellow senior scientists are present to consume their attention. Overall, these more informal events allow students to become familiar with the way national and international conferences are conducted and make these events less intimidating when they attend a regular conference later on in their career.

These student-led events explicitly aim to facilitate the exchange of information via informal discussions and often via oral and poster presentations. Participants have the opportunity to summarize the main themes of their work in short talks, demanding an objective look at their own research project. The presenters have to take one step back from their daily work and think about the overall project and goals. They learn how to clearly explain their specific topic, which is a skill required to be able to set up collaborations and initiate multidisciplinary projects later on.

Conferences organized by students enable more students to present their work, as opposed to the stricter selection criteria enforced at formal, regular conferences that limit the extent of student participation. Presenting their work encourages the students to defend their findings in the company of their peers, building on their presentation and communication skills in an environment that feels more relaxed than regular conferences. Students are able to receive valuable feedback and comments from peers, which serves to help improve their research and as a preliminary step towards the peer review publication process.

## Examples of Successful Student Conferences

Conferences organized by different RSGs across the world have been highly successful, with each catering for the specific needs of the region in which it was held. For example, RSG Southern and Eastern Africa assisted in bringing together approximately 150 students and seasoned scientists from around the African continent to Cape Town, South Africa to share their work at the ISCB Africa ASBCB (African Society for Bioinformatics and Computational Biology) Conference on Bioinformatics and the Young Researchers Forum (YRF) preceding it [6]. The focus of this conference was on infectious diseases relevant to Africa, like HIV and tuberculosis. The YRF was a collaborative event with students trained in human genetics, which offered bioinformatics students the chance to present their work prior to the conference, at no extra cost, and had the added benefit of interaction with a different audience than the main conference.

RSGs have also attempted the interesting and challenging concept of virtual conferencing. A virtual conference is designed to replicate the experience of participating in a conference but it is carried out wholly over the internet. The benefit of this approach is that it can bring together a large group of people who are geographically distant from each other at low cost. Virtual conferencing vastly reduces organizational expenses, is easy on student finances, excludes high registration fees, and is a convenient mode of attending an event. Users are able to log into a virtual conference from their office, their favorite spot at home, or even their couch. The main advantage comes from the quality of the information exchanged between groups at various locations globally. A scientist from Caltech or MIT can conveniently give a keynote talk for a conference in India without actually having to travel there through the use of different web conferencing tools. RSGs from Africa were the frontrunners in organizing virtual conferences in bioinformatics and computational biology [7]. Known as Afbix (later editions as Bifx), the first virtual conference had three successful editions, garnering great enthusiasm from the region. The virtual con ferences were of great benefit to these regions, as they had limited grants and income sources to sponsor physical conferences.

RSG Argentina has also played a pivotal role in facilitating informal social interaction and networking by organizing student meetings adjacent to the Second and Third Argentine Congress on Bioinformatics and Computational Biology conferences [8]. The First National Meeting for Bioinformatics and Computational Biology Students, held two days before the third Argentinian conference, included an array of activities taught by advanced PhD students and researchers with the aim of providing undergraduate- and/or graduate-level students from different backgrounds with useful tools for their own research projects.

## Challenges for the Organisers

Aside from the successes RSGs have had in hosting past events, there have also been a few hurdles along the way. A perpetual challenge in the organization of any event is the acquisition of sufficient funds. However, many RSGs have discovered that funding can be found by approaching various scientific companies and organizations that have an interest in student capacity development. In addition, a few useful strategies have been to use existing infrastructure from national bioinformatics centres to reduce costs and to leverage local contacts to gain support in kind, like access to meeting rooms from the local university.

Once funding has been acquired, the next hurdle has usually been making sure the conference is well advertised. This has been overcome by reaching out to students in different organizations and on different mailing lists. We found that organizing the student conference close to the time of a larger conference ensures a good attendance. To increase the interest of students and make them even more excited about the conference, prizes have also been offered in both the oral and poster presentation categories.

## Conclusion

Meeting and overcoming the challenges mentioned above have been highly rewarding both for the participants and the organizers. The students involved in the organization of conferences gain experience in the process of initiating, coordinating, and following through on an event of this magnitude [9]. For students attending the conference, it is a chance to network, review and be reviewed by their peers, and make plans to work with new people with similar research interests. This form of social education engages the audience through participation and interaction, which is a vital component of learning.

**About the Authors**

The authors have been involved in several aspects of the ISCB Student Council and its Regional Student Groups. **Kavisha Ramdayal** was president of RSG-Southern-Africa (2007–2012). **Miranda Stobbe** was a co-founder and secretary of RSG-Netherlands (2008–2012). **Tarun Mishra** was president of RSG-India (2010–2012). **Magali Michaut** was co-founder and president (2008–2010) of RSG-France and served on its Board of Directors (2008–2013). She was also co-founder, secretary (2009), and president (2010–2011) of RSG-Europe and served as secretary for the ISCB Student Council (2009).
